# Polymer–Metal Hybrid Composites: An Overview of the Role of Metal Architecture

**DOI:** 10.3390/ma19091678

**Published:** 2026-04-22

**Authors:** Ana Pavlovic, Carlo Santulli, Cristiano Fragassa

**Affiliations:** 1 Department of Industrial Engineering, University of Bologna, 40136 Bologna, Italy; ana.pavlovic@unibo.it; 2 School of Science and Technology, University of Camerino, 62032 Camerino, Italy; carlo.santulli@unicam.it

**Keywords:** polymer–metal hybrid composites, metallic architectures, metallic inserts, interface engineering, adhesion mechanisms, additive manufacturing, hybrid processing, mechanical performance, multifunctional composites, structural design

## Abstract

Polymer–metal hybrid composites (PMHCs) represent an emerging class of materials that combine the lightweight processability of polymers with the structural and functional advantages of metals. Recent advances in material design and manufacturing have shifted attention from traditional particulate or fibrous reinforcement toward metallic architectures—continuous, architected, or topologically optimized metallic networks intentionally embedded within polymer matrices. These metallic architectures play a key role in defining the composite’s global performance, influencing stiffness, energy absorption, failure mechanisms, and multifunctional properties such as electrical or thermal conductivity. This review examines how the geometry, connectivity, and topology of metallic reinforcements govern mechanical behavior and functional responses in PMHCs. Emphasis is placed on the interplay between architecture and interface design, including surface modification strategies and mechanical interlocking phenomena. Furthermore, the paper discusses the contribution of additive manufacturing technologies in enabling complex metallic architectures and hybrid processing routes. By integrating structural, interfacial, and manufacturing perspectives, this review develops a coherent framework for understanding how metallic architecture drives the evolution of PMHCs toward multifunctional and design-driven engineering applications. The analysis of the literature consistently indicates that architectural configuration—rather than material selection alone—represents the primary factor governing multifunctional performance.

## 1. Introduction

### 1.1. Background and Context

The demand for lightweight, high-performance, multifunctional materials has been steadily increasing across sectors such as aerospace, automotive, electronics, and energy systems. These industries continually seek materials that can deliver structural strength and stiffness without compromising manufacturability or increasing weight [1,2].

In response to these needs, composite materials—defined as systems combining two or more distinct constituent phases at the material or structural level to achieve properties unattainable by any single material alone—have increasingly been adopted as a key materials solution.

Within this framework, hybridization has emerged as a modern design strategy, referring to the deliberate combination of different material classes, phases, or architectures within a composite system in order to expand the range of achievable property combinations beyond conventional single-reinforcement or single-matrix approaches.

**Figure 1:**
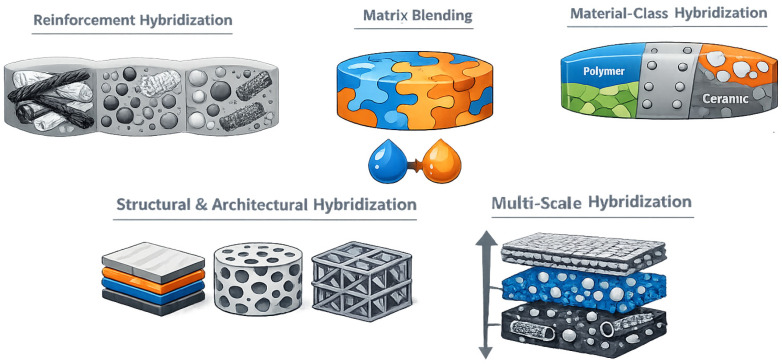
Schematic representation of hybridization classes in composite materials.

Hybridization can be implemented at multiple levels of the material system, each offering distinct opportunities for tailoring performance and functionality, including the following (Figure 1):

- Reinforcement type, through the combination of different reinforcing phases, such as multiple fiber types (e.g., glass and carbon fibers), fibers and particulate fillers, or micro- and nanoscale reinforcements, to balance stiffness, strength, damage tolerance, and multifunctional properties.
- Matrix composition, by blending two or more polymer matrices or by combining matrices with different chemical or physical characteristics, enabling the tuning of toughness, thermal stability, and processing behavior.
- Material classes, via the integration of fundamentally dissimilar materials—such as polymers, metals, or ceramics—within a single composite system, allowing complementary mechanical and functional attributes to be combined.
- Structural and architectural configuration, including hybrid laminates, layered systems, graded structures, or architected reinforcements, where geometry and topology are deliberately designed to control load transfer, anisotropy, and failure mechanisms.
- Length and hierarchical scales, by coupling reinforcements acting at different dimensional scales (nano-, micro-, and macroscale), thereby extending hybridization from the constituent level to multi-scale material architectures.

In the literature, some ambiguity exists in the terminology used to describe hybrid polymer-based composites, and caution is therefore required when interpreting existing classifications. In particular, the terms polymer hybrid composites and hybrid polymer composites are often used to describe distinct, yet partially overlapping, material concepts [3]. Polymer hybrid composites generally refer to systems in which a single polymer matrix is reinforced with multiple types of fillers or reinforcements, such as fibers, particulates, or functional nanofillers. In this case, the hybridization design approach is primarily materials-based and aims at exploiting the synergistic effects between different reinforcing phases embedded within the polymer matrix. Hybrid polymer composites, by contrast, typically denote materials in which two or more polymers are combined or blended, with or without additional fillers, to tailor mechanical or functional properties through polymer–polymer interactions rather than through structural reinforcement.

**Figure 2:**
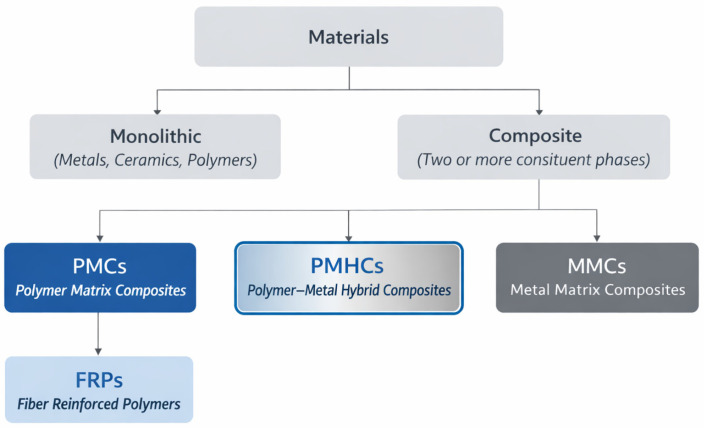
Positioning of PMHCs within material classification.

Within this context, PMHCs have emerged as an independent and technologically relevant class of advanced materials [4] in which the metallic phase is intentionally integrated within a polymer matrix to provide both structural load paths and multifunctional responses, with overall performance governed by the coupled effects of architecture, interface design, and processing route (Figure 2).

PMHCs combine the low density, corrosion resistance, and process versatility of polymers with the mechanical robustness, electrical and thermal conductivity, and durability of metals [5]. The characteristic combination of these properties enables PMHCs to bridge the performance gap between pure polymer composites and metallic structures, offering engineers design flexibility and enhanced functionality. PMHCs should not be confused with hybrid metal matrix composites (MMCs), where the load-bearing matrix is metallic and the hybridization involves different types of ceramic or fibrous reinforcements rather than a polymeric matrix [6].

Unlike conventional fiber- or particle-reinforced polymer composites, where the reinforcement phase typically consists of discrete and mechanically discontinuous elements, PMHCs [4] incorporate a continuous or architected metallic phase that provides a structural backbone within the polymer matrix [7]. This design allows for efficient load transfer and the creation of structural networks capable of sustaining complex stresses. Moreover, metallic inclusions can introduce electrical, thermal, or magnetic properties, making PMHCs suitable for structural–functional integration, where a single component can serve both as a load-bearing element and as a conductive or heat-dissipating unit.

In recent years, advances in interface engineering and hybrid additive manufacturing have enabled controlled integration of metallic reinforcements in complex geometries. Consequently, PMHCs have transitioned from niche experimental systems to an emerging platform for next-generation multifunctional materials.

While this distinction is useful for classifying filler-based and polymer-blend systems, it does not adequately capture the characteristics of PMHCs with continuous or architected metallic phases addressed in the present review. In these systems, the metallic component is not merely a dispersed filler nor a secondary modifier, but acts as a structurally and functionally active architecture, providing continuous load paths, controlled topology, and multifunctional capabilities. As such, PMHCs based on metallic architectures represent a distinct design paradigm that cannot be fully described within conventional polymer hybrid or hybrid polymer composite classifications.

### 1.2. Historical Evolution of Polymer–Metal Hybrid Composites

**Figure 3:**
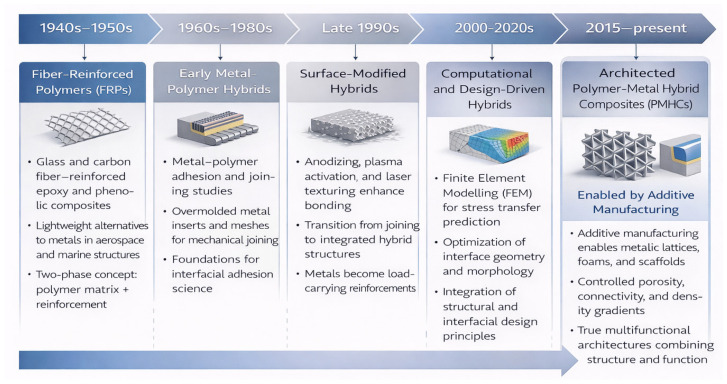
Evolution of composite materials leading to PMHCs.

The origins of PMHCs are deeply rooted in the historical development of composite materials (Figure 3).

During the 1940s and 1950s, fiber-reinforced polymers (FRPs) such as glass fiber-reinforced epoxy and phenolic composites revolutionized materials engineering by providing lightweight alternatives to metallic alloys in aerospace and marine applications (e.g., [8,9,10]). These materials demonstrated the potential of combining two distinct phases—a polymer matrix and a reinforcement—to achieve property synergies. However, early FRPs were limited in architecture: their reinforcements were either one-dimensional (fibers) or zero-dimensional (particles). These systems achieved high specific strength but offered little flexibility in tailoring the load distribution and failure mechanisms within the composite structure.

In parallel, metal–polymer adhesion studies emerged in the 1960s–1980s, motivated by the need for reliable joining between dissimilar materials in automotive and consumer products. Early “hybrid” concepts were limited to overmolded metal inserts, where a metal sheet or wire mesh was embedded into a molded polymer for enhanced joining strength. These assemblies, though not yet true composites, established the foundation for understanding interfacial adhesion between metals and polymers.

By the late 1990s, improved surface modification techniques—including anodizing, plasma activation, laser structuring, and micro-mechanical texturing—enabled the controlled enhancement of interfacial bonding [11]. These developments paved the way for second-generation polymer–metal hybrids, where the metallic component was not merely a joining element but part of a load-carrying hybrid structure.

In the 2000s, finite element modeling (FEM) began to provide predictive insights into stress transfer across metal–polymer interfaces, further motivating the optimization of insert geometry and surface morphology.

The past decades (2010–2025) have witnessed the rise in third-generation PMHCs, characterized by architected metallic phases designed with topological precision. The concurrent maturation of additive manufacturing (AM)—notably laser powder bed fusion (LPBF), electron beam melting (EBM), and directed energy deposition (DED)—has enabled the fabrication of metallic lattices, foams, and porous scaffolds with tunable porosity, connectivity, and density gradients. When infiltrated or co-processed with polymer matrices, these structures produce hybrid composites with unprecedented combinations of mechanical and functional properties [12].

Thus, PMHCs today represent the convergence of three key critical research areas:

1. Architectural design of hybrid composite structures;
2. Interfacial engineering and adhesion mechanisms;
3. Advanced manufacturing and topology-driven optimization.

This convergence defines a rapidly expanding field bridging materials science, mechanical engineering, and design innovation.

### 1.3. From Reinforcements to Architectures

**Figure 4:**
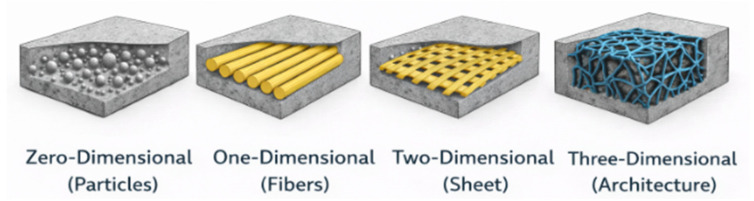
Schematic of reinforcement dimensionalities in composites: from particles to architectures.

The concept of reinforcement in composites has evolved from simple inclusion toward designed architecture. This evolution can be interpreted in terms of increasing reinforcement dimensionality and structural continuity: from zero-dimensional particles to one-dimensional fibers, to two-dimensional sheets or laminates, and finally to fully three-dimensional architected metallic networks (Figure 4).

In conventional composites, the reinforcing phase is typically discrete and mechanically discontinuous; particles and short fibers primarily act as local stiffness or strength enhancers, while even continuous fibers mainly provide directional reinforcement without forming a fully interconnected structural framework. In these systems, load transfer is governed by interfacial shear along discrete inclusions, and the reinforcement remains morphologically subordinate to the matrix.

By contrast, the transition toward architected metallic phases represents not merely an increase in dimensionality, but a conceptual shift. Three-dimensional metallic architectures introduce continuous and topology-controlled load paths within the polymer matrix, enabling stress redistribution, progressive damage mechanisms, and multifunctional integration at the structural scale. Rather than acting as passive inclusions, these metallic networks function as active skeletal frameworks whose geometry, connectivity, and spatial arrangement become primary design variables.

In this sense, PMHCs based on metallic architectures move beyond traditional reinforcement concepts: the metallic phase is intentionally designed as an active, continuous load path or as a multifunctional skeleton within the polymer matrix, capable of simultaneously governing stiffness, energy absorption, electrical or thermal transport properties.

**Figure 5:**
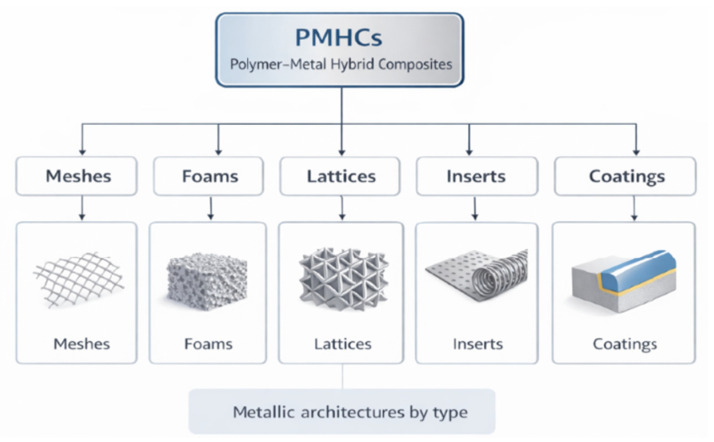
PMHCs classified by metallic architecture type.

The term “metallic architecture” characterizing PMHCs traditionally encompasses a wide variety of structural motifs, including the following (Figure 5):

- Metal meshes and expanded sheets, which provide in-plane stiffness and fracture bridging.
- Metal foams and porous scaffolds, offering energy absorption and damping [13].
- Lattice and cellular structures, produced via AM, enabling controlled topology and gradient functionality.
- Wire networks, inserts and woven metallic fabrics, designed for flexibility and anisotropy.
- Metallic coatings and surface metallization, which provide conductive, protective, or functional layers on polymer substrates.

These architectures determine not only the composite’s mechanical performance but also its functional response, such as thermal and electrical conductivity, EMI shielding, and structural damping. Importantly, the topology, connectivity, and surface morphology of the metallic reinforcement strongly influence how stresses are distributed and how damage propagates under static or dynamic loads.

Recent research has shown that architecture-driven properties can even surpass those of conventional FRPs. For example, metallic lattices infiltrated with polymer matrices can achieve energy absorption densities comparable to aluminum foams while maintaining superior impact resistance. Similarly, hybrid structures combining conductive metallic skeletons and insulating polymers enable lightweight, multifunctional components suitable for smart structures, sensors, and lightweight electromagnetic shields.

In this sense, PMHCs represent not merely a variation in existing composite systems but a paradigm shifts toward functionally architected materials, where geometry and interface design are as important as composition.

### 1.4. Interface and Processing Challenges

Despite their advantages, PMHCs present significant challenges associated with the metal–polymer interface. The main challenge arises from the strong mechanical, thermal, and chemical mismatch between the two phases. 

Metals are stiff, conductive, and crystalline, while polymers are soft, insulating, and amorphous. This mismatch gives rise to stress concentrations, residual thermal strains, and poor adhesion if the interface is not properly engineered.

Several adhesion mechanisms have been identified:

- Mechanical interlocking, achieved by creating micro- or nanoscale surface roughness through laser texturing, sandblasting, or anodization.
- Chemical bonding, via coupling agents (e.g., silanes, phosphonic acids) or reactive surface coatings.
- Physical interactions, including van der Waals forces and localized diffusion bonding in high-temperature processes.

The design of the metal surface and the selection of the polymer matrix must therefore be approached in an integrated manner. Surface chemistry and topography directly affect not only adhesion strength but also fatigue and environmental durability.

On the manufacturing side, the integration of metal and polymer components can be achieved through the following:

- Overmolding and insert injection molding, widely used in automotive parts.
- Resin impregnation or transfer molding, suited for metallic foams and wire meshes.
- Co-curing or lamination, often applied to hybrid sheet structures.
- HAM, enabling the creation of highly controlled architectures.

A comprehensive review of joining technologies for polymer–metal hybrid structures highlighted injection overmolding as a key industrial process in which interfacial strength is governed by surface pre-treatments, thermal history, and polymer flow-induced mechanical interlocking [14]. However, combining metals and polymers in a single process chain introduces complexities such as thermal mismatch during curing, residual stress accumulation, and dimensional instability. Moreover, early studies on fiber metal laminates have already shown that curing-induced residual stresses arise from thermo-mechanical mismatch between metallic layers and polymer-based composites, significantly influencing interfacial integrity and structural performance [15]. Although several joining strategies for polymer–metal hybrid structures have been established, their performance remains strongly dependent on surface preparation, thermal history, and residual stresses, as discussed in existing reviews [16]. Recent progress in hybrid additive manufacturing (HAM)—combining metal 3D printing with polymer printing or infiltration—has opened new pathways for producing PMHCs with precise control of the metal–polymer interface, although scalability and repeatability remain open challenges [17].

### 1.5. Need for a Comprehensive Review

Given the rapid diversification of research on polymer–metal hybrid systems, there is a pressing need for a comprehensive and integrative review that examines not only individual case studies but also the underlying design principles connecting architecture, interface, and performance.

Most of the existing literature focuses on narrow aspects: fiber–metal laminates, bonding mechanisms, or manufacturing processes. What remains lacking is a unified framework that captures the role of metallic architecture as a design variable influencing both mechanical and multifunctional behavior.

This review therefore aims to provide a critical synthesis of the state of the art, analyzing how different metallic architectures—meshes, foams, lattices, and inserts—affect the following:

- Load transfer and stiffness;
- Failure and damage tolerance;
- Interfacial adhesion, and;
- Functional responses (thermal, electrical, and electromagnetic).

Moreover, this review discusses the role of AM and topology optimization as key enabling technologies for the next generation of PMHCs. By explicitly linking structure, processing routes, and performance, it outlines future directions toward design-driven PMHCs capable of meeting the structural and multifunctional demands of modern engineering applications. Beyond material selection and processing considerations alone, growing evidence indicates that the architecture of the metallic phase constitutes a primary design variable in PMHCs. Rather than acting as a secondary reinforcement or merely as a joining feature, metallic architectures govern load transfer, failure mechanisms, and multifunctional response, thereby defining the overall structural behavior of PMHC systems. Accordingly, this review adopts an architecture-centered perspective, integrating structural, interfacial, and manufacturing aspects within a unified design framework.

It is structured as follows. Section 2 describes the methodological framework adopted for the literature review, including the selection criteria and classification strategy used to organize existing studies. Section 3 presents a structured analysis of PMHCs according to increasing architectural complexity, progressing from continuous metallic reinforcements to porous structures, lattice-based designs, and HAM approaches. Within each category, the relationships between architecture, interfacial behavior, mechanical performance, and multifunctional response are critically discussed. The review concludes by synthesizing these findings into an architecture-centered perspective, highlighting key trends, limitations, and future research directions for the design of next-generation PMHCs.

## 2. Materials and Methods

### 2.1. Review of General Aspects

This review aims to provide a comprehensive overview of recent advances in PMHCs, with a specific focus on the influence of metallic architectures and inserts on mechanical, functional, and interfacial performance. The objective is to synthesize experimental, numerical, and theoretical findings from the literature to clarify how metallic structures—ranging from meshes and foams to architected lattices—affect load transfer mechanisms, failure behavior, and multifunctional properties. The review also seeks to identify trends in manufacturing techniques, particularly the integration of additive and hybrid processing routes, and to highlight open challenges and future research opportunities in the design of architected polymer–metal systems.

### 2.2. Literature Search Strategy

**Figure 6:**
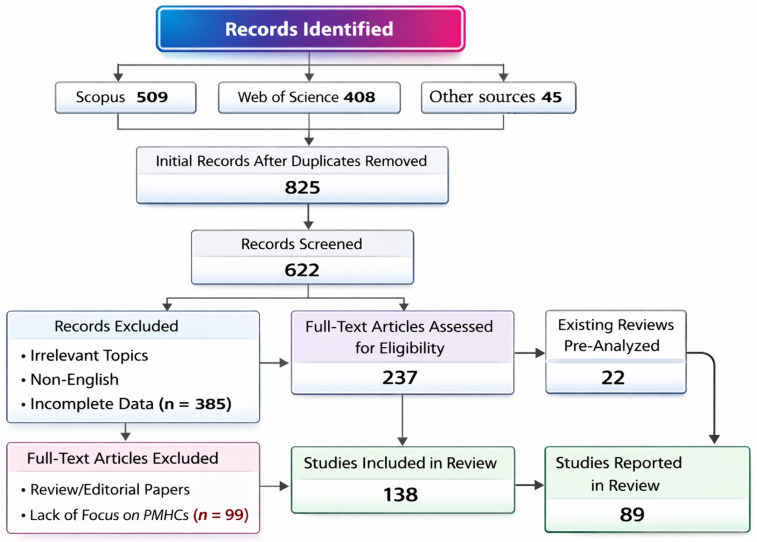
Flowchart of the literature search and selection process for PMHCs analysis.

A structured literature search was conducted following principles similar to the *PRISMA* (Preferred Reporting Items for Systematic Reviews and Meta-Analyses) framework, adapted to materials science (Figure 6).

Relevant publications were retrieved from major scientific databases, including the following:

- Scopus;
- Web of Science;
- ScienceDirect;
- SciSpace;
- MDPI Open Access;
- IEEE Xplore;
- SpringerLink, and;
- Google Scholar (especially for cross-verification).

The literature search was conducted between October and December 2025 and therefore includes publications available up to the end of 2025. The search mainly covered the period 2015–2025, ensuring inclusion of both foundational studies and recent developments in advanced manufacturing and architecture-driven composite design, while also allowing for the inclusion of selected earlier works. Primary search terms included combinations of the following: “polymer–metal hybrid composites”, “metallic architectures”, “metallic inserts”, “hybrid composites”, “additive manufacturing”, “interfacial adhesion”, “mechanical performance”, and “multifunctional behavior”. Boolean operators (AND, OR) and truncations were used to broaden coverage. Example: (“polymer–metal hybrid composite*” OR “metal–polymer composite*”) AND (“architecture*” OR “insert*” OR “reinforcement*” OR “additive manufacturing”).

### 2.3. Selection Criteria

Publications were included if they

- Reported experimental, numerical, or analytical studies involving polymer–metal hybrid or architected composite systems.
- Addressed interfacial behavior, structural design, or multifunctional properties (mechanical, electrical, thermal, or electromagnetic).
- Provided quantitative and/or comparative data allowing evaluation of architecture–property relationships.
- Were peer-reviewed journal papers, conference proceedings, or book chapters.

Publications were excluded in the cases of

- Studies limited to simple metal–polymer adhesion tests without composite formation.
- Papers on non-architected or purely particulate nanocomposites.
- Duplicates, non-English or non-peer-reviewed publications.

### 2.4. Data Extraction and Organization

For each selected publication, key information was extracted, considered and, when possible, categorized according to the following:

- Type of metallic architecture (mesh, foam, lattice, perforated sheet, wire network, printed insert).
- Matrix type (thermoplastic, thermosetting, elastomeric).
- Interface modification or treatment (mechanical interlocking, coating, plasma, chemical coupling).
- Manufacturing route (injection molding, resin transfer molding, overmolding, additive manufacturing).
- Evaluated properties (tensile strength, stiffness, impact, fatigue, conductivity, EMI shielding, etc.).

Data were summarized to highlight structure–property–process relationships. Where possible, dimensionless parameters (e.g., relative density, reinforcement volume fraction, stiffness ratio) were used to enable cross-comparison among studies.

### 2.5. Classification Framework

To facilitate the present systematic analysis, the selected works were grouped into thematic categories:

- Continuous metallic reinforcements (meshes, sheets, perforated foils);
- Interfacial engineering and bonding mechanisms;
- Porous and foam-based architectures;
- Lattice- and topology-optimized structures;
- HAM approaches;
- Functional performance (electrical, thermal, and EMI properties).

Each group was analyzed in terms of structural design principles, interface behavior, processing–performance relationships, and emerging trends.

### 2.6. Critical Evaluation and Synthesis

The collected data were synthesized through comparative analysis and cross-referenced interpretation. Emphasis was placed on the following:

- Quantifying the influence of architectural parameters (porosity, connectivity, topology) on mechanical and multifunctional outcomes.
- Identifying scaling relationships and mechanisms recurrent across material systems.
- Assessing the compatibility between metal and polymer phases and the impact of interfacial treatments.
- Mapping the evolution of hybrid manufacturing routes (e.g., metal 3D printing + polymer infiltration).

When conflicting findings were encountered, they were analyzed in relation to differences in architectural configuration, material combination, interfacial treatment, or processing route, rather than treated as isolated discrepancies. Trends were considered consistent when similar structure–property relationships were reported across independent studies with comparable architectural features, even when constituent materials differed.

Statistical or meta-analytical methods were not applied, as the review aims to integrate diverse datasets through qualitative and mechanistic reasoning.

### 2.7. Overview of Existing Reviews and Identified Gaps

As part of the methodological framework of this review, a comparative analysis of existing review papers and state-of-the-art studies was carried out to better position the present work within the broader context of hybrid composite research.

In recent years, several review papers have addressed various aspects of hybrid and multifunctional composite systems, reflecting the growing industrial and academic interest in combining materials. However, these studies differ considerably in scope and focus.

A large part of them primarily discussed polymer-based hybrid systems, typically reinforced with combinations of fibers, particulates, or nanofillers rather than metallic structures (as in [1,2,5]). These contributions provided essential background on hybridization mechanisms and property enhancement, without exploring aspects such as the integration of metallic phases as continuous reinforcements. More focused (and recent) works have examined PMHCs from a manufacturing and interfacial perspective, with emphasis on joining techniques such as injection overmolding, co-curing, and metal surface treatments ([4,11]). These reviews offer valuable insights into adhesion mechanisms, surface engineering, and industrial processing strategies for hybrid structures. Other studies have also reviewed surface modification and coating methods for improving metal–polymer bonding [18], as well as electrochemical and metallization routes relevant to HAM [19]. Complementary analyses on MMCs and architected cellular materials (e.g., [6,13]) have significantly contributed to the understanding of topology-controlled mechanical behavior, yet these studies focus on metallic systems rather than polymer–metal hybrids. Similarly, the emerging literature on additively manufactured hybrid composites has highlighted the potential of combining metal lattices with polymers, but a systematic synthesis linking architecture, interface design, and multifunctional response remains absent [17]. From a functional standpoint, reviews addressing electrical and dielectric behavior in polymer–metal nanocomposites [20] and conductive foams [13] have provided insight into conductivity mechanisms, yet they lack a unified perspective linking architecture to performance. Meanwhile, reviews dedicated to modeling and cohesive zone prediction of metal–polymer interfaces [21] contribute valuable tools for interfacial design. Some contributions adopt an application-driven perspective, such as book chapters addressing PMHCs for functional or biomedical uses, rather than a systematic analysis of metallic architectures and load-bearing behavior [22]. Similarly, recent reviews have addressed additively manufactured polymer–metal scaffolds, primarily in biomedical contexts, with emphasis on porosity, fabrication routes, and biocompatibility rather than on architecture-driven structural performance [23].

**Figure 7:**
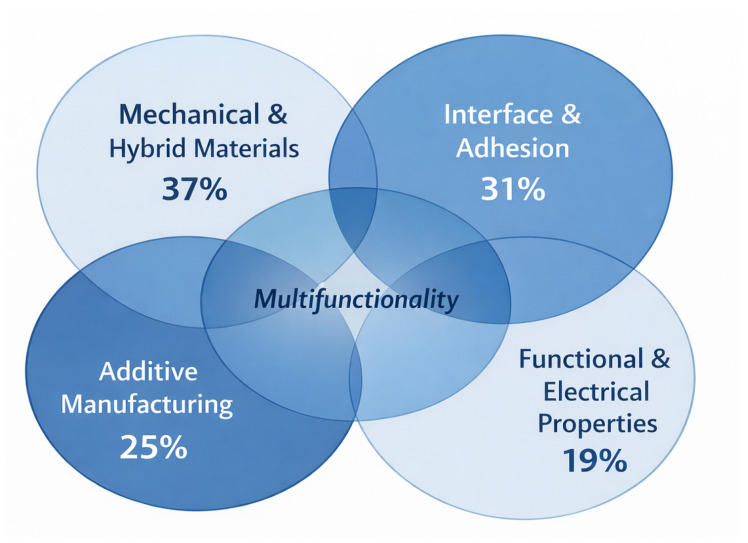
Thematic domains and overlaps among existing review studies on PMHCs.

Nevertheless, these investigations are rarely integrated with architectural considerations. Their discussion often remains property-oriented or process-oriented, with limited attention to the architectural and topological aspects that define mechanical performance and multifunctionality. As a result, most of these studies tend to cluster around the domains of Interface and Adhesion and Additive Manufacturing, with overlaps toward Mechanical and Hybrid Materials and Functional and Electrical Properties (Figure 7).

The present review aims to fill that gap by integrating insights from structural, processing, and design-oriented research, providing a comprehensive overview of PMHCs from the perspective of metallic architecture (Table 1).

Overall, while existing reviews provide valuable insights into hybridization concepts, joining technologies, AM processes, or application-specific functionalities, none of them explicitly address metallic architecture as a governing design variable linking structure, interface, and performance in polymer–metal hybrid composites. This lack of an architecture-centered framework motivates the present review and defines its specific scope. 

### 2.8. AI Use Statement

AI-based software tools were used to support specific tasks within the research workflow. SciSpace (v2026) and Google Scholar were employed for literature search and initial screening, while OpenAI GPT-5.2 and DeepL Translator (v2026) were used for language refinement and stylistic editing. AI-assisted image tools (e.g., Canva and Remove.bg) were applied to enhance the visual quality of figures originally produced by the authors. All AI-generated outputs were critically reviewed, validated, and, where necessary, substantially revised by the authors. No AI tools were used for data generation, analysis, or the derivation of scientific results.

## 3. Results and Analysis

The following sections treat metallic architecture as a primary structural design variable in polymer–metal hybrid composites, rather than as an ordinary reinforcement morphology or a process-dependent feature. The analysis is therefore organized by increasing architectural complexity, highlighting how topology, connectivity, and geometry of the metallic phase directly govern mechanical behavior, interfacial response, and multifunctional performance.

Based on the classification and selection criteria defined in the previous section, the following results summarize and critically discuss the current state of the art in polymer–metal hybrid composites. The analysis is structured by increasing structural complexity—from continuous metallic inserts to porous, lattice, and fully architected designs—emphasizing how each configuration influences mechanical performance, interface behavior, and functional properties. This approach allows for a comprehensive understanding of how metallic architecture shapes the overall behavior of hybrid composites.

### 3.1. Continuous Metallic Reinforcements (Meshes, Sheets, Perforated Foils)

Among the earliest and most intuitive approaches to PMHCs, the use of continuous metallic reinforcements—such as meshes, perforated foils, and thin sheets—represents a highly effective strategy for combining the strength and stiffness of metals with the ductility, light weight, and formability of polymers. In these systems, the metallic phase acts as a continuous load-bearing skeleton that distributes stresses efficiently across the polymeric matrix, preventing local failure and improving overall mechanical stability. Unlike particles or short fibers, continuous metallic elements provide uninterrupted load paths. This enhances stiffness and delays cracking or delamination. In practice, these reinforcements can take the form of wire meshes, expanded metal foils, or thin metallic sheets with controlled perforation patterns. The open geometry of meshes allows the molten or softened polymer to infiltrate the metallic structure during processing, producing a form of mechanical interlocking that contributes to improved interfacial adhesion. Perforated foils, on the other hand, offer a balance between stiffness and polymer bonding, as the distribution and size of holes govern the degree of polymer penetration and load transfer. Thin metallic sheets are often used in metal–polymer–metal (MPM) sandwich structures, which provide excellent bending resistance and are particularly suited to forming-intensive processes in the automotive and transportation sectors (as discussed in [24]). Manufacturing routes for PMHCs with continuous metallic reinforcements typically rely on techniques such as co-lamination, hot pressing, injection overmolding, or polymer infiltration of pre-formed metallic inserts. During processing, the polymer fills the interstices or perforations of the metallic phase, forming a mechanical bond that complements any chemical adhesion developed at the interface. The effectiveness of this process depends strongly on the surface condition of the metal—its roughness, oxide layer thickness, and wettability—as well as on the viscosity and thermal stability of the polymer matrix. Properly optimized, these systems display remarkable pseudo-ductility, combining metal-like strength with polymer-like energy absorption and crack resistance.

#### 3.1.1. Structural Behavior and Performance

Representative studies summarized in Table 2 demonstrate the diversity of continuous reinforcement configurations explored in the past decade. Metal–polymer–metal sandwiches reinforced with local metallic meshes showed improved formability in deep-drawing and bending operations. The inclusion of metallic inlays enabled plastic deformation without premature delamination, proving the concept of locally reinforced hybrid sheets for forming-intensive applications [25]. Hybrid composite architectures reinforced with wire mesh networks also demonstrated that continuous metallic frameworks significantly increase tensile stiffness and load-carrying capacity, while maintaining reasonable formability [7]. Aluminum mesh-reinforced polymer composites incorporating natural bamboo fibers further highlighted the influence of mesh aperture size and stacking sequence, revealing up to 25% gains in tensile strength and substantial improvements in strain-to-failure when the mesh–matrix interface was optimized [26]. Beyond mechanical reinforcement, continuous metallic architectures have also been exploited for functional enhancement. Metal mesh-reinforced CFRP laminates demonstrated improved electromagnetic interference (EMI) shielding together with increased bending stiffness, confirming the multifunctional potential of PMHCs [27]. The embedded copper or stainless-steel meshes, in fact, not only enhanced shielding effectiveness but also increased bending stiffness, demonstrating the feasibility of multifunctional PMHCs with both structural and electrical performance. Similarly, moreover, under high strain-rate loading, metal-layered hybrid composites exhibited enhanced impact resistance and energy absorption, particularly in crash and ballistic scenarios [28]. Together, these studies underline how continuous metallic reinforcements can be tailored in geometry, topology, and composition to meet diverse functional and mechanical demands. The degree of polymer infiltration, metal surface roughness, and thermal compatibility between phases emerge as dominant factors controlling the composite’s overall performance.

#### 3.1.2. Interfacial Adhesion and Failure Mechanisms

The primary advantage of continuous reinforcements lies in the efficient stress transfer they enable across the metal–polymer interface. The metallic phase, being continuous, acts as a load-distributing skeleton, mitigating local stress concentrations within the polymer and thereby delaying failure initiation. At the same time, the polymer phase provides damping capacity, flexibility, and corrosion protection, balancing the brittleness typically associated with metallic lattices.

From a design standpoint, three key parameters govern performance:

(1) Metallic topology and porosity—mesh size, hole geometry, and sheet perforation ratio directly affect polymer infiltration, interfacial adhesion, and stiffness;
(2) Interface quality and bonding mechanism—the success of continuous reinforcements depends heavily on the adhesion between the metal and polymer, often improved by surface texturing or chemical treatment;
(3) Processing conditions—molding temperature, pressure, and polymer viscosity determine how effectively the polymer wets and bonds the metallic substrate, influencing residual stresses and dimensional stability.

Notably, mechanical performance is strongly anisotropic: in-plane tensile and bending stiffness increase with metal continuity, whereas interlaminar shear strength depends on polymer adhesion. Optimization therefore requires balancing metal continuity for load transfer with polymer penetration for interfacial integrity. The fracture behavior of environmentally friendly fiber metal laminates (FMLs) showed that continuous aluminum layers significantly increase fracture toughness through crack bridging and crack arrest mechanisms. The hybrid architecture promotes progressive damage rather than catastrophic failure, confirming the decisive role of metallic continuity in controlling fracture propagation in PMHC laminates.

### 3.2. Interfacial Engineering and Bonding Mechanisms

The performance and long-term reliability of PMHCs depend critically on the strength, stability, and nature of the interface that binds the metallic and polymeric constituents. Regardless of the type of metallic architecture—whether continuous meshes, foils, or cellular structures—the efficiency of load transfer across the hybrid system is governed by the quality of adhesion at the metal–polymer boundary. This interfacial region represents a complex mechanical and chemical transition zone, where differences in stiffness, surface energy, and thermal expansion must be reconciled. Recent research has focused on developing both experimental and modeling-based approaches to characterize and optimize bonding mechanisms, addressing the challenges of delamination, fatigue, and failure propagation. For instance, experimental peel and shear tests on polymer–metal hybrids confirmed that interfacial adhesion is strongly governed by metallic surface morphology, microscale cavities enabling mechanical interlocking, and the degree of wetting achieved during overmolding, with small variations in surface preparation producing substantial differences in fatigue and shear strength [29].

#### 3.2.1. Experimental Approaches to Interfacial Strengthening

Several studies have explored how surface engineering and joining processes can enhance metal–polymer adhesion. An innovative joining route for metal–polymer–metal sandwich panels based on wire-mesh interlayers placed at the skin–core interface was proposed [30]. The metal sheets are locally joined to the wire mesh via resistance welding, and vacuum hot pressing is then used to infuse the thermoplastic core into the mesh, generating a robust mechanical interlocking at the interface. The resulting panels exhibited a marked increase in interface strength (e.g., peel performance) compared with adhesive-bonded joints reported in the literature, highlighting the synergy between engineered interfacial architecture and load transfer capability—particularly relevant under cyclic loading conditions in automotive and aerospace applications. An ultrasonic vibration-assisted hot-pressing route using a Mg–Zn–Al eutectic alloy interlayer enabled metallurgical bonding in CFRP/Mg hybrid laminates, addressing the adhesion challenges between carbon fiber composites and magnesium [31]. Mechanical testing (e.g., DCB/3ENF) and tensile characterization showed markedly improved interlaminar performance and overall mechanical response compared with conventional epoxy-bonded Mg-FMLs, highlighting the potential of low-temperature, solid/near-solid interlayers to strengthen the CFRP/Mg interface. This study represents a milestone in understanding chemical–mechanical coupling at the interface of dissimilar materials. Among the emerging joining techniques, laser-based methods have shown exceptional precision and cleanliness in forming localized polymer–metal bonds with minimal heat-affected zones, making them attractive for lightweight structural applications [32]. Electromagnetic riveting (EMR) has been proposed as a solid-state joining technique for polymer–metal hybrid structures, enabling mechanical interlocking without significant thermal input [18]. This technique employs pulsed electromagnetic forces to plastically deform metallic sheets at high velocity, generating mechanical interlocks with polymer or composite layers without adhesives or significant thermal input. Its applicability to aerospace-grade multi-material assemblies demonstrates the potential of EMR as a complementary route to laser joining for high-integrity PMHC interfaces.

#### 3.2.2. Modeling and Simulation of Interfacial Behavior

Beyond experimental optimization, several studies have approached interfacial phenomena from a computational mechanics perspective. A finite element (FE) model was developed to predict crack initiation and propagation at metal–polymer interfaces [33]. Their model incorporated fracture mechanics principles and cohesive zone elements to capture interfacial debonding, enabling quantitative prediction of local energy release rates and failure propagation under complex loading. This approach bridges the gap between experimental data and predictive design, providing a foundation for *virtual prototyping* of hybrid joints. A constitutive model for polymer–metal interfaces based on cohesive zone modeling (CZM) was formulated to describe interfacial behavior and failure evolution [34]. Their work described adhesive–cohesive transitions and failure propagation mechanisms through calibrated traction–separation laws. By capturing both elastic and damage evolution behaviors, the model enables accurate prediction of interfacial performance under various stress states. This modeling framework can guide the optimization of surface treatment and process parameters, reducing the experimental burden in PMHC development. In situ experimental monitoring combined with finite element analysis was used to investigate stress evolution within the interfacial region. Strain gauges and embedded sensors captured local stress gradients during fatigue cycles and correlated them with debonding initiation, identifying fatigue regimes dominated by cohesive or adhesive failure and improving understanding of durability under cyclic loading [35].

Further studies on hybrid composite–metal systems analyzed the influence of process-induced residual stresses on interfacial damage propagation and monitored the interface using embedded optical fiber sensors, providing experimental insight into durability-critical mechanisms relevant to PMHCs [36].

#### 3.2.3. Mechanical Coupling and Interlocking Mechanisms

While surface modification and modeling dominate recent research, some studies revisit more mechanically driven strategies for achieving robust bonding (Table 3). Mechanical coupling systems joining composite and metallic components through hybrid fastening and preload distribution have shown that optimized joint geometry and controlled preload significantly improve load transfer, delay interfacial failure, and ensure stable performance under shear and bending. These findings highlight the key role of mechanical design variables—beyond interfacial chemistry alone—in ensuring interfacial reliability in PMHC-related systems [37]. Direct metal–polymer joining between advanced high-strength steel and PA6 using laser surface texturing demonstrated that joint strength and failure mode strongly depend on the applied texturing pattern [38]. A mechanical interlocking approach based on localized “mechanical nuggets” has been proposed to join metal–polymer sandwich sheets [39]. This technique creates discrete, ductile metallic–polymeric interlocks that resist pull-out and fatigue damage without relying on traditional adhesives. The joints exhibited strong adhesion and excellent recyclability, offering a sustainable alternative to adhesive-based or thermally cured hybrid interfaces. The work demonstrates the growing shift toward adhesive-free joining strategies that favor circular design and end-of-life disassembly of hybrid structures.

### 3.3. Porous and Foam-Based Architectures

Porous and foam-based metallic architectures represent a versatile and rapidly evolving class of PMHCs. In these systems, a metallic foam or open-cell scaffold serves as a continuous, lightweight, and permeable skeleton that can be infiltrated or coated with a polymeric phase, leading to a fully interpenetrating or semi-interpenetrating network. These hybrids combine the stiffness and conductivity of metals with the damping and energy absorption of polymers, resulting in lightweight and multifunctional materials. Because of their complex microstructure, they are especially attractive for impact mitigation, vibration damping, lightweight structures, and smart functional applications such as sensing, filtration, or energy absorption. For instance, a dedicated infiltration system for manufacturing metal foams and metal–polymer interpenetrating composites demonstrated that precise control of pressure gradients and melt-flow rates enables the formation of highly uniform porous structures, minimizing trapped-air defects and improving mechanical stability under compressive loads [40].

#### 3.3.1. Structural Concept and Processing Strategies

The key idea behind porous and foam-based PMHCs is to combine an open-cell metallic network with a polymeric matrix that infiltrates its voids, forming an interpenetrating microstructure. The metallic component typically consists of aluminum, copper, nickel, or stainless-steel foams, fabricated through techniques such as powder sintering, replication, gas expansion, or casting with space holders. The polymer is introduced by melt infiltration, resin impregnation, in situ polymerization, or adhesive coating. This design enables three-dimensional connectivity of both phases: the metal ensures load transfer and structural stability, while the polymer absorbs energy and provides damping or flexibility. An essential advantage of this approach is that both materials remain continuous, avoiding the weak interfacial bonds often seen in layered systems. However, the success of these hybrids strongly depends on pore morphology, polymer viscosity, and wetting behavior during processing. Uniform polymer infiltration without voids or debonding is critical for achieving consistent mechanical performance and durability. Foundational work on the production and stability of metallic foams has demonstrated how pore morphology and structural uniformity critically determine the mechanical stability and energy absorption efficiency of foam-based systems [41]. Early studies introduced the Advanced Pore Morphology (APM) concept, combining aluminum foams with polymeric phases for automotive crash structures [42]. This work demonstrated that controlling pore shape and distribution is key to maximizing both strength and energy absorption. Similarly, open-cell aluminum foams infiltrated with thermoplastics were employed to investigate vibration and damping behavior, highlighting how the interfacial region between polymer and metal dictates dynamic stiffness and energy dissipation [43,44].

#### 3.3.2. Mechanical Behavior and Energy Absorption

Porous and foam-based PMHCs display a characteristic combination of high specific stiffness, compressive strength, and damping capacity. The metallic skeleton provides a load-carrying backbone, while the polymeric phase contributes to strain recovery and energy dissipation through viscoelastic deformation. Under compressive or impact loading, the metallic ligaments deform plastically, while the polymer fills help stabilize the cellular structure, preventing premature buckling and collapse. In this context, it was demonstrated that additively manufactured steel lattices filled with polymeric phases behave as true PMHCs, where the metallic architecture governs load transfer while the polymer infiltration enhances energy absorption and stabilizes progressive collapse mechanisms under compression [45]. This dual-phase behavior was modeled using a tetrakaidekahedral finite element framework, showing that the arrangement and aspect ratio of metal ligaments strongly affect the macroscopic stiffness and collapse mode [46]. It was also demonstrated that polymer infiltration shifts the deformation mechanism from brittle fracture toward ductile, energy-absorbing failure. These phenomena were later summarized, noting that foam morphology—pore size, shape, and distribution—is the dominant factor governing energy absorption, largely independent of the specific polymer type [47]. These findings confirm that tuning the microstructure of the metallic foam enables precise control of the balance between stiffness, toughness, and damping.

#### 3.3.3. Interfacial Behavior and Dynamic Properties

The interface between the metal skeleton and the infiltrated polymer plays a central role in defining the hybrid’s mechanical and acoustic behavior. Adhesion is typically mechanical, arising from polymer anchoring within the porous network, though surface oxidation or roughening can enhance wetting and bonding. The dynamic response of foam-based PMHCs has been widely studied in the context of vibration isolation, impact damping, and NVH (noise–vibration–harshness) applications. The interfacial damping capacity in metal–polymer hybrid foams was shown to increase with polymer filling fraction and bonding quality at the metal–polymer interface. Experimental modal analyses further revealed that hybrid foams outperform pure metal or pure polymer foams in terms of damping ratio and loss factor, supporting their potential for vibration control in automotive and aerospace applications [43,44].

#### 3.3.4. Functional Porous Hybrids and Emerging Applications

Beyond mechanical performance, porous metal–polymer hybrids have evolved into functional materials with electrical, electrochemical, and sensing capabilities (Table 4). Cu-coated melamine foams have been fabricated as elastic and conductive scaffolds for microbial electrochemical systems, demonstrating stable long-term electrochemical operation [48]. However, the polymeric phase serves primarily as a sacrificial or supporting template for the formation of conductive architectures, rather than as a load-bearing matrix, and the material design is driven by electrochemical performance rather than mechanical integrity, interface mechanics, or multifunctional structural behavior typical of PMHCs. Polymer-coated metallic foams have been developed combining high elasticity, electrical conductivity, and pressure sensitivity, enabling their use in flexible tactile sensors and smart material applications [49]. At smaller scales, MOF–polymer hybrid systems bridge the gap between structural and nanostructured porous materials. The design of MOF–polymer composites has been reviewed, outlining synthetic strategies to achieve hierarchical porosity and selective permeability. However, in these systems the metal phase does not function as a structural reinforcement, and hybridization is primarily aimed at chemical functionality rather than the mechanical or multifunctional behavior typical of PMHC architectures [50]. Nanoporous MOF–polymer hybrids have been explored in biomedical contexts, highlighting their tunable porosity, surface chemistry, and potential for drug delivery and biosensing applications. However, these systems are optimized for chemical and biological functionality rather than for structural or multifunctional polymer–metal hybrid performance and therefore differ fundamentally from the PMHC architectures considered here [51]. At the same time, taken together, these studies highlight parallel research directions in functional porous hybrid materials, where the interplay between structure and chemistry defines new application spaces beyond the scope of structurally oriented PMHCs. Finally, architected interpenetrating metallic phases produced through hybrid manufacturing exhibited high specific stiffness and markedly improved damage tolerance, as the continuous dual-phase network distributes mechanical loads synergistically and restricts crack propagation even under elevated stress levels [52].

### 3.4. Lattice- and Topology-Optimized Structures

The advent of AM and computational design has transformed how hybrid polymer–metal systems are conceived and fabricated. Among these innovations, lattice- and topology-optimized architectures stand out as a new frontier for achieving high stiffness-to-weight ratios, tailored energy absorption, and multifunctionality. Unlike random foams or continuous reinforcements, these architectures rely on periodic or hierarchical unit-cell designs, often generated through topology optimization algorithms and subsequently realized via AM processes such as laser powder bed fusion (LPBF), fused filament fabrication (FFF), or HAM combining both metal and polymer materials. In these systems, the metallic lattice acts as a high-strength skeleton capable of bearing mechanical and thermal loads, while the polymer phase provides damping, flexibility, or electrical insulation. Through geometry-driven design, properties can be spatially tuned—allowing local stiffness gradients, energy absorption zones, or conductive pathways within the same structure.

#### 3.4.1. Early Applications of Topology Optimization in PMHCs

The earliest systematic approach to topology optimization for polymer–metal hybrids was proposed by [53]. This pioneering work at Clemson University and BMW integrated size, shape, and topology optimization for automotive body-in-white structures. By coupling FE-based optimization with manufacturability constraints related to injection overmolding, they demonstrated that the topology of metallic inserts within polymer components could be optimized for both stiffness and weight reduction, paving the way for the current generation of design-for-hybridization methods.

Further experiments also demonstrated that applying a controlled metallic coating onto polymer meso-lattice structures substantially increases both stiffness and collapse strength, as the metallic shell redistributes stresses through the lattice nodes and struts and delays localized buckling [54].

#### 3.4.2. Hybrid Metal–Composite Lattice Systems

A major step forward was made with Di Caprio et al. (2019) [55], who introduced hybrid metal/composite lattice structures specifically tailored for additive and filament winding processes. Their numerical tool, developed using ANSYS and Mode Frontier, enabled automatic generation and optimization of metallic lattice unit cells integrated with composite shells. The optimized architectures significantly improved buckling resistance and load transfer, demonstrating the potential of lattice cores in aerospace-grade PMHC components. Similarly, topology optimization combined with metallization of polymer lattices has been used to develop compression-resistant hybrid structures inspired by biological designs such as cuttlebone. The resulting topology-optimized lattices achieved a specific modulus exceeding 5400 MPa·kg^−1^ and an energy absorption efficiency of 78%, demonstrating that topologically guided microarchitectures can outperform conventional random foams [56]. Topology-optimized metallic cellular frameworks produced via AM showed significantly enhanced energy absorption capacity when coupled with polymeric matrices, displaying a highly stable progressive collapse and an extended stress plateau driven by the engineered deformation pathways of the optimized lattice [57].

#### 3.4.3. Multi-Scale and Multi-Material Design Strategies

As the field evolved, researchers began merging topology optimization with multi-scale and multi-material modeling. A two-step design method combining classical topology optimization with lattice-based refinement has been developed to generate solid–lattice hybrid structures. These architectures achieved 20–30% weight reduction while maintaining mechanical integrity, demonstrating effectiveness in aerospace applications such as frames and brackets [58]. Multi-material topology optimization has been applied to composite–metal hybrid aircraft structures by combining the Moving Morphable Components (MMCs) and level-set approaches. The results demonstrated that computational optimization could generate feasible and manufacturable geometries for real-world hybrid systems. 

#### 3.4.4. Hybrid Additive Manufacturing and Experimental Validation

Recent advances have established hybrid additive manufacturing (HAM) as a key enabler for PMHC lattices. A compression overmolding process integrating laser powder bed-printed maraging steel lattices with additively manufactured carbon fiber-reinforced polyamide preforms has been developed to produce structurally integrated hybrid systems [59]. This approach yielded structures with high stiffness, tensile strength, and damage tolerance, validated through experimental tensile and microscopy analyses. The work demonstrates the potential of combining AM precision with polymer infiltration or overmolding to create robust hybrid lattices. Complementing this, fully architected polymer–metal lattice composites were developed via hybrid additive manufacturing, combining vat photopolymerization of polymer lattices with subsequent Ni-P/Cu electroplating to form continuous metallic networks, resulting in lightweight multifunctional structures exhibiting high ductility, electrical conductivity, and mechanical performance comparable to pure copper, with demonstrated applicability to UAV components [19].

#### 3.4.5. Topology-Optimized and Impact-Resistant Lattices

Beyond mechanical optimization, several studies have focused on energy absorption and failure control (Table 5). Fiber-reinforced polymer lattice beams designed through anisotropic topology optimization have been investigated, showing that the introduction of a perimeter constraint enhances post-failure toughness and prevents shear banding, providing valuable insights for damage-tolerant lattice design [60]. The Bidirectional Evolutionary Structural Optimization (BESO) algorithm has been applied to design novel lattice topologies (CompIED, ShRComp) with exceptional impact resistance. These architectures exhibited superior energy absorption under compression compared with conventional TPMS or truss lattices [61]. A multi-material topology optimization method has been introduced to design lattice–stiffener hybrid cores for composite sandwich panels, ensuring uniform distribution and improved in-plane load transfer [62].

### 3.5. Hybrid Additive Manufacturing Approaches

HAM represents one of the most transformative developments in the design and production of PMHCs. Recent classifications of AM and HAM technologies provide the technological framework enabling the fabrication of PMHCs with controlled architectures [63].

In contrast to traditional manufacturing routes—such as insert molding, resin infiltration, or mechanical joining—HAM integrates multiple fabrication modalities within a single platform, enabling the sequential or concurrent deposition of metallic and polymeric phases. This hybridization can occur through direct energy-based metal deposition (e.g., laser powder bed fusion, directed energy deposition, Additive Friction Stir Processing) followed by polymer infiltration, overmolding, or photopolymerization steps, effectively combining the structural stiffness of metals with the damping and formability of polymers.

As highlighted by [64,65], HAM technologies bridge the gap between metallic additive processes and polymeric additive techniques, offering previously unattainable interfacial control and design freedom. These methods allow for local reinforcement, multi-material gradient architectures, and on-demand tailoring of thermal–mechanical interfaces. Foundational frameworks by [6,12] established the conceptual basis of *architected cellular materials*, defining topological and hierarchical parameters—such as porosity, connectivity, and relative density—that have since been extended to PMHCs. Within this context, hybrid AM is no longer viewed merely as a joining method, but as a multi-scale material design strategy where the architecture, composition, and interface are simultaneously optimized. This has opened the path toward *integrated digital manufacturing*—from topology optimization to layer-by-layer hybrid fabrication—capable of generating complex metallic skeletons and polymer matrices with embedded functional pathways.

#### Experimental and Computational Advances

Recent studies demonstrate that coupling metal and polymer additive processes enables structurally integrated, multifunctional architectures (Table 6). A compression overmolding process combining laser powder bed-printed maraging steel lattices with carbon fiber-reinforced polyamide shells achieved strong metal–polymer adhesion and high stiffness-to-weight ratios, validated through finite element analysis [59]. A dual-phase hybrid additive manufacturing technique integrating vat photopolymerization with electroless and electroplating of Ni–P/Cu coatings has been developed to produce polymer–metal lattice composites with metallic conductivity and ductility, extending PMHC applications to UAV and robotics components [19]. Fused deposition modeling (FDM) has been demonstrated as a direct hybrid joining route, enabling localized polymer deposition onto metallic substrates and achieving strong interfacial bonding without additional adhesives [66].

From a design perspective, topology optimization has been applied to improve post-failure behavior and load distribution in hybrid lattices and sandwich cores. The introduction of perimeter constraints and lattice–stiffener co-design was shown to mitigate shear banding and ensure more uniform stiffness distribution [60,62]. The Bidirectional Evolutionary Structural Optimization (BESO) approach has been further applied to generate novel lattice topologies (CompIED, ShRComp) with enhanced impact resistance and energy absorption under compression [61].

In biomedical-oriented systems, several studies have demonstrated the additive co-fabrication of porous polymer–metal scaffolds in which a load-bearing metallic phase is combined with a polymeric matrix to provide compliance and enhanced biocompatibility [67,68]. In these hybrid architectures, AM enables precise control of scaffold porosity and phase connectivity, allowing the metallic component to contribute stiffness and structural support while the polymer phase accommodates deformation and biological integration. Although these approaches were primarily developed for bone tissue engineering applications, the same HAM strategies are directly transferable to structural PMHCs, particularly for lightweight components and energy-absorbing structures where controlled porosity, damage tolerance, and multifunctionality are required.

### 3.6. Functional Performance (Electrical, Thermal, and EMI Properties)

Beyond their structural integrity and lightweight design, PMHCs are increasingly being developed to deliver multifunctional performance, integrating electrical conductivity, thermal management, and electromagnetic interference (EMI) shielding within a single material system. The evolution from purely mechanical reinforcement toward functionally active hybrid architectures is driven by the growing demand for high-performance materials in aerospace, electronics, energy storage, and communication technologies. These functional properties originate from the synergistic interaction between the metallic conductive domains and the polymeric insulating or dissipative phases, whose interfacial engineering governs charge transport, heat flow, and electromagnetic attenuation. In this context, recent studies have demonstrated that the careful design of filler architecture, interfacial coupling, and HAM strategies can enable PMHCs to achieve structural and functional integration at unprecedented levels. In general, continuous 3D networks typically favor stable bulk conductivity and volumetric EMI attenuation, whereas layered sheets or embedded meshes mainly promote directional, in-plane transport and reflection-based shielding. Porous or lattice-based structures may further modulate heat flow and electromagnetic dissipation through topology-controlled pathways.

#### 3.6.1. Electrical Performance

Electrical functionality in PMHCs arises from the formation of continuous or semi-continuous conductive pathways through metallic or hybrid filler networks. These conductive domains can be introduced either through embedded metallic meshes, nanoparticles, or hybrid carbon–metal structures, which together enable tunable conductivity and dielectric response across broad frequency ranges. Interfacial dispersion of metallic nanoparticles and conductive polymers within hybrid matrices has been shown to significantly enhance both electrical conductivity and permittivity, enabling stable charge transport and reduced dielectric losses [20]. Similarly, PANI/graphite hybrid composites have been synthesized, achieving enhanced electrical conductivity together with a 72 MPa increase in tensile strength, demonstrating that mechanical robustness can coexist with conductive performance [21].

A lossy dielectric mechanism has been demonstrated in natural rubber/acetylene black PMHCs, providing moderate EMI shielding (≈20 dB) together with improved mechanical compliance, suitable for flexible electronic applications [69]. Thermoplastic starch (TPS) composites filled with waste iron filings have been investigated, showing that metallic waste particles can form electrically conductive pathways within biodegradable polymer matrices. The results highlighted the importance of filler dispersion and percolation thresholds in controlling conductivity while maintaining acceptable mechanical integrity, confirming that electrical performance in PMHCs strongly depends on the microstructural organization of the metallic phase [70].

Exceptional multifunctionality has been achieved in interface–layup hybrid CF/epoxy laminates incorporating Fe nanoparticles and MnO_2_ interlayers, resulting in a synergistic enhancement of electrical conductivity, EMI shielding effectiveness, and interlaminar shear strength [70]. These approaches underscore that hybrid electrical performance in PMHCs depends not only on filler type but also on interfacial coupling, dispersion homogeneity, and anisotropic architecture design.

#### 3.6.2. Thermal Performance

Thermal performance is a critical aspect for PMHCs in aerospace, automotive, and electronic applications, where rapid heat dissipation and structural stability are required. The incorporation of metallic or hybrid fillers with high intrinsic thermal conductivity (e.g., Cu, Fe–Si–Al, graphene, Al_2_O_3_, or SiC) significantly enhances heat transfer pathways within the polymer matrix. Three-dimensional interconnected metal–polymer architectures and surface-modified Fe–Si–Al hybrid composites have been shown to achieve thermal conductivities exceeding 6 W/mK while maintaining lightweight characteristics [71,72]. Similarly, Cu hollow bead–epoxy PMHCs have been produced combining low density (~1 g/cm^3^) with high thermal conductivity (7 W/mK), establishing an early benchmark for multifunctional heat-dissipating composites [73].

Recent computational and modeling studies have advanced the understanding of heat percolation and interfacial scattering effects. Micromechanical models integrating micro- and nanofillers (SiO_2_–graphene) predicted up to a threefold improvement in thermal conductivity [74]. Excluded-volume optimization in hybrid composites has been shown to enhance thermal percolation by up to 20%, highlighting the benefit of multi-scale filler distribution [75]. Moreover, emerging high-thermal conductivity polymers have been reviewed, identifying filler alignment, covalent coupling, and surface functionalization as key strategies to exceed 10 W/mK—a significant milestone for structural PMHCs [76]. These insights collectively indicate that hybrid AM and interfacial engineering are essential for next-generation thermally efficient polymer–metal systems.

#### 3.6.3. Electromagnetic Interference (EMI) Shielding Performance

EMI shielding represents one of the most prominent functional properties of PMHCs, driven by the need to protect sensitive electronics and communication systems from electromagnetic pollution (Table 7). The mechanism typically combines reflection (from conductive fillers), absorption (from magnetic or lossy phases), and multiple scattering (via porous or layered structures) [77,78]. A 41 dB EMI shielding effectiveness (SE) in flexible CNT/Fe_3_O_4_/PP composites was achieved, integrating magnetic absorption with thermal regulation [79]. PPS matrices filled with CNTs, graphene nanoplatelets, and carbon fibers achieved dual functionality, combining EMI shielding effectiveness of 50–68 dB with enhanced thermal conductivity (7 W/mK) [80]. CF/epoxy–MnO_2_/Fe hybrid laminates have demonstrated EMI shielding effectiveness of up to 70 dB, showing that interface–layup design can create anisotropic conductive pathways beneficial for both electromagnetic shielding and heat dissipation [70].

At a microstructural level, the combination of metallic, carbonaceous, and magnetic fillers (e.g., Fe_3_O_4_, Ni, Cu, graphite) provides synergistic EMI attenuation through impedance matching and multireflection. Some reviews highlight the evolution from simple filler-dispersed systems toward architected hybrid networks, including additive-manufactured or electroless-plated lattices [77,81]. These designs enable broadband EMI attenuation while maintaining structural integrity and light weight. Thus, EMI performance in PMHCs is increasingly viewed as a system-level property, emerging from the cooperative effects of electrical, magnetic, and structural hybridization.

## 4. Discussion and Perspectives

Several key insights emerge from the review when adopting an architecture-driven perspective on PMHCs. The discussion focuses on the underlying mechanisms and design trade-offs governing structural performance, interfacial efficiency, and multifunctional behavior across different classes of metallic architectures. Rather than revisiting individual studies, it emphasizes recurring trends and cross-cutting concepts, highlighting how geometry, topology, and processing routes collectively shape the response of PMHCs. In this context, Table 8 provides a comparative and architecture-oriented synthesis of PMHCs, highlighting how different classes of metallic architectures translate into distinct performance profiles and design trade-offs. It emphasizes that no single solution simultaneously maximizes all attributes. Continuous and planar metallic inserts excel in load transfer and industrial scalability, porous and foam-based architectures provide superior energy absorption and damping, while lattice- and topology-optimized structures enable unprecedented stiffness-to-weight ratios at the expense of manufacturability. Conversely, metallized polymer architectures primarily address functional integration rather than structural reinforcement.

### 4.1. Continuous Metallic Reinforcements: Structural and Functional Trade-Offs

Recent research is extending continuous metallic reinforcement design toward multifunctional hybrids. Beyond structural enhancement, embedded metallic sheets or meshes serve additional purposes: electromagnetic shielding (as demonstrated in [27]), thermal management and heat dissipation in electronics housing, and damage sensing or self-heating functionalities when metallic networks are instrumented electrically. Advanced additive manufacturing techniques now enable the fabrication of patterned and graded metallic reinforcements, allowing topologically optimized stiffness and density gradients that further expand the design possibilities of PMHCs. In short, continuous metallic reinforcements remain a foundational category within polymer–metal hybrid composites, bridging traditional metal–polymer laminates and architected metamaterial hybrids. Their success lies in the synergy between geometric continuity, interfacial adhesion, and manufacturability. Future developments are expected to exploit hierarchical design—where metallic sheets, perforations, and meshes are patterned in a functionally graded manner—to achieve lightweight, formable, and multifunctional PMHCs tailored for next-generation structural applications.

### 4.2. Interfacial Engineering: Interface-Controlled Performance in PMHCs

Across the most representative studies dealing with the interface-controlled performance of PMHCs, several consistent trends emerge:

1. Synergistic bonding mechanisms: The strongest and most durable PMHCs rely on the combination of mechanical anchoring (through roughness or interlocks) and chemical bonding (through surface functionalization or oxide formation).
2. Multi-scale modeling: Constitutive and cohesive models (Hirsch, Pan) now enable predictive simulation of debonding, fatigue, and failure propagation, paving the way for design-by-simulation approaches.
3. Process–structure–property integration: The effectiveness of interfacial engineering depends not only on material selection but also on processing conditions—molding temperature, polymer viscosity, surface preparation—that dictate interface morphology.
4. Sustainability and recyclability: New joining approaches show that mechanical interlocking can replace adhesives, reducing environmental impact and simplifying recycling of hybrid assemblies [39].

### 4.3. Porous and Foam-Based Architectures: Energy Absorption and Damping

Porous and foam-based architectures in polymer–metal hybrids provide a compelling pathway to achieve lightweight, energy-absorbing, and multifunctional materials. Their key advantages lie in the interpenetrating nature of the metallic and polymeric phases, enabling synergistic combinations of strength, damping, and flexibility. At the same time, challenges remain in controlling pore morphology, polymer infiltration uniformity, and interfacial adhesion, especially for large-scale manufacturing. Recent progress in additive manufacturing, foaming control, and surface functionalization is opening new directions toward architected porous PMHCs with graded properties and tailored functionalities.

### 4.4. Lattice- and Topology-Optimized Architectures: Geometry-Driven Performance

Lattice- and topology-optimized PMHCs represent the culmination of geometry-driven material design, where structure, composition, and functionality are intertwined at multiple scales. By integrating computational optimization, multi-material additive manufacturing, and microstructural tailoring, these hybrids achieve performance levels previously unattainable by conventional composites.

Current research trends point toward the following:

- Graded lattice architectures with continuous metal–polymer transitions.
- Bioinspired designs using natural geometries (e.g., cuttlebone, trabecular bone).
- Integrated simulation–manufacturing pipelines for defect-free hybrid production.

As the field matures, topology-optimized PMHCs are poised to redefine lightweight structural design, impact protection, and multifunctional components across aerospace, robotics, and automotive sectors.

### 4.5. Hybrid Additive Manufacturing: Process–Architecture Integration

Across the reviewed studies, HAM is converging toward a unified design–manufacturing paradigm in which the metal–polymer interface, lattice topology, and functional properties are co-optimized. The main emerging trends can be summarized as follows:

- Integrated multi-material design: HAM enables the simultaneous printing and bonding of metals and polymers, creating architectures with graded stiffness, tunable damping, and localized conductivity.
- Topological and hierarchical optimization: Combining computational topology tools (BESO, MMC, and lattice refinement) with AM allows spatial tuning of hybrid architectures for specific load paths or thermal functions (as in [61] or [62]).
- Additive Friction Stir Processing (AFSP): Offers a route to metallurgically bond polymer–metal interfaces under solid-state conditions, minimizing residual stresses and enabling strong, defect-free interfaces. Solid-state approaches derived from friction-stir processing have been increasingly explored to tailor near-surface microstructures and to enhance bonding quality in hybrid builds, while mitigating typical fusion-based issues such as porosity, oxidation and residual stresses [83].
- Cross-disciplinary applications: Biomedical AM scaffolds [84] and architected metamaterials [12,16] exemplify how HAM concepts can be extended beyond aerospace and automotive sectors, toward energy systems, robotics, and soft electronics.

Despite rapid progress, challenges remain in thermal mismatch management, scalability, and repeatability of hybrid processes. Achieving consistent metallurgical–chemical bonding between dissimilar phases requires advances in interfacial surface engineering, real-time process monitoring, and closed-loop manufacturing control. Nonetheless, the reviewed body of work demonstrates that HAM has established itself as a foundational technology for the next generation of functionally graded, topology-optimized, and multifunctional polymer–metal hybrid composites.

### 4.6. Functional Integration: Electrical, Thermal, and EMI Perspectives

The state of the art confirms that PMHCs can be engineered to achieve simultaneous improvements in electrical conductivity (10^−3^–10^2^ S/cm), thermal conductivity (3–10 W/mK), and EMI shielding effectiveness (20–100 dB), without compromising mechanical strength. The integration of hierarchical fillers, interfacial coupling agents, and layer-engineered architectures enables multifunctionality once considered incompatible in single-phase materials. Emerging HAM techniques and 3D network design are now bridging the gap between structural and functional performance, allowing custom-tailored PMHCs for aerospace, electronics, and space applications. In summary, PMHCs have transitioned from purely structural materials to multifunctional, architected systems, capable of simultaneous mechanical reinforcement, heat management, and electromagnetic protection. Future developments will depend on optimizing filler architecture, hybrid process integration, and scalable manufacturing routes to achieve both functional and economic viability.

### 4.7. New Architectural Paradigms

Finally, it is fundamental to consider that, beyond the archetypes mentioned before (Figure 5), recent studies have introduced several emerging architectural paradigms, including perforated or patterned metallic inserts for enhanced adhesion, hierarchical hybrids combining meshes and lattices, and functionally graded metallic frameworks with spatially varying density. Additionally, AM has enabled the creation of interpenetrating metal–polymer networks and metallized polymer skeletons, bridging structural and functional design in next-generation PMHCs.

A more extended and systematic classification of metallic architectures, integrating both conventional and emerging structural typologies, is provided in Table 9.

### 4.8. Secondary Raw Materials and Circular Design

PMHCs may also offer interesting opportunities in the context of circular material strategies. Their architectural nature—where metallic and polymeric phases are intentionally combined in structured configurations—can facilitate the integration of secondary raw materials, including recycled or bio-based polymers (e.g., reprocessed thermoplastics or bio-derived matrices) and reclaimed metallic forms (e.g., scrap sheets, expanded meshes, or machining residues).

Recycled thermoplastic feedstocks, such as reprocessed polypropylene, polyethylene, or bio-based polymer blends, can be employed as matrix materials, particularly in systems where processing routes such as compression molding or infiltration tolerate moderate variability in rheological behavior. Similarly, recycled metallic forms—including scrap sheets, expanded meshes, machining residues, or recovered metal particles—may serve as reinforcing or conductive phases within hybrid architectures. In certain configurations, continuous metallic meshes or structured inserts can accommodate dimensional variability more effectively than particulate reinforcements, reducing sensitivity to geometric inconsistencies.

The use of secondary materials may influence mechanical and functional performance depending on purity, surface oxidation, and processing history. Recycled polymers can exhibit reduced molecular weight or altered viscoelastic response, while recycled metals may present surface irregularities that affect interfacial adhesion and load transfer. Nevertheless, appropriate surface treatment, architectural continuity, and controlled interface design can mitigate part of these limitations.

From a design perspective, PMHCs may thus represent a flexible platform for integrating secondary raw materials, balancing structural performance with resource efficiency. However, challenges remain in terms of property reproducibility, quality control, and long-term durability, particularly in applications requiring high reliability.

## 5. Conclusions

The present review recognizes metallic architecture as a primary design variable in PMHCs. Across continuous, porous, and lattice-based systems, the topology and connectivity of the metallic phase consistently emerge as dominant factors controlling stiffness, damage tolerance, energy absorption, and multifunctionality. Consequently, future developments in PMHCs should prioritize architecture-driven design strategies, supported by advanced manufacturing and interface engineering, rather than relying solely on material selection or processing optimization.

While recent studies have advanced joining and HAM routes, most of them remain process-driven, focusing on isolated steps (overmolding, laser joining, electromagnetic riveting, or FDM deposition) rather than integrated design approaches. Similarly, the growing research on surface metallization and graded coatings has provided localized improvements in bonding and conductivity, but these have not yet been coupled with the hierarchical control of metallic geometry that modern AM techniques can provide.

Functionally, the available literature demonstrates that metallic networks within polymers can enhance electrical conductivity, EMI shielding, damping, and thermal management, yet quantitative structure–property relationships are rarely established. The multifunctionality of PMHCs is often described qualitatively, with insufficient coupling between experimental data and predictive modeling. Existing modeling approaches, including cohesive zone and finite element formulations, are powerful but typically calibrated for flat or simplified interfaces, not for architected metallic geometries with multi-scale features. Addressing this limitation requires unified simulation frameworks capable of capturing topology-dependent adhesion and failure mechanisms, validated by experimental data on graded and additively manufactured systems.

Therefore, this review highlights the need for a multi-scale design framework where metallic architecture acts as the primary variable connecting process parameters, interfacial chemistry, and multifunctional performance.

In short, the following key evidence emerges from the present analysis:

1. PMHCs are defined not simply by combining metals and polymers, but by intentionally designing the metallic architecture as the main structural and functional element.
2. PMHCs can be grouped according to their metallic architecture: laminates (FMLs), meshes and sheets, foams and porous networks, lattice/topology-optimized structures, and interpenetrating systems.
3. Mechanical performance is mainly controlled by metallic topology and connectivity, which govern load transfer, stiffness, energy absorption, and failure modes.
4. Interfacial quality is critical: surface treatment and mechanical interlocking strongly affect strength and durability.
5. Manufacturing routes (lamination, infiltration, overmolding, additive manufacturing) directly influence architectural accuracy and interface integrity.
6. Under impact, fatigue, or compression, PMHCs show architecture-driven mechanisms such as crack arrest and progressive damage redistribution.
7. Compared to single-phase metals or polymers, PMHCs provide higher specific stiffness and added multifunctionality (i.e., thermal, electrical, and EMI applications).
8. The field still lacks standardized testing, reliable multi-scale models, and clear quantitative links between architecture and performance.
9. AM expands design freedom, enabling controlled metallic skeletons and shifting hybrid design toward geometry-driven optimization.

**Figure 8:**
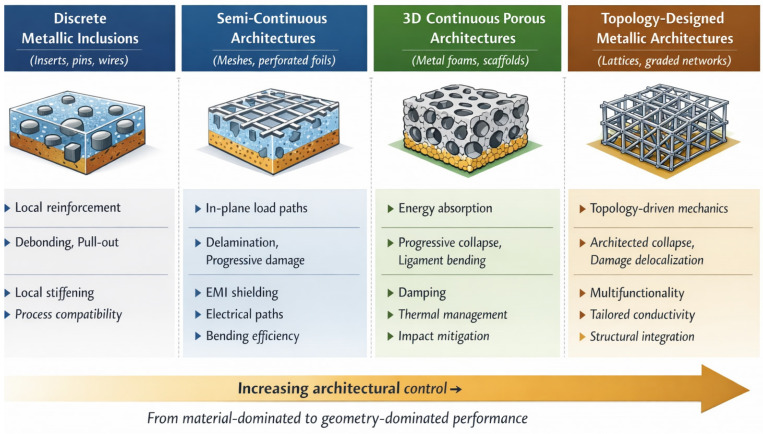
Architecture-driven classification of PMHCs.

Specifically, an architecture-driven classification of PMHCs can be summarized in Figure 8. It shows how different metallic architectures embedded in a polymer matrix govern load transfer mechanisms, damage modes, and multifunctional performance. The figure highlights a progressive transition from discrete metallic inclusions to semi-continuous networks, 3D porous structures, and fully topology-designed metallic architectures, emphasizing the shift from material-dominated to geometry-dominated composite behavior.

This architecture-driven perspective positions PMHCs as a new class of designable hybrid materials, where geometry, interface, and functionality are co-engineered to achieve application-specific structural and multifunctional performance.

Future research priorities emerge directly from the identified gaps and prioritize the following:

(i) The development of integrated design–manufacturing–characterization workflows combining topology optimization, metal AM, and polymer infiltration;
(ii) The establishment of quantitative design rules linking architecture metrics (porosity, cell shape, ligament aspect ratio) to mechanical and functional outputs;
(iii) The use of hybrid numerical–experimental models to predict interfacial evolution and failure across scales.

Such an integrative approach will enable the rational design of PMHCs with tailored architectures, optimized interfaces, and predictable multifunctionality—transforming them from empirical material systems into designable structural–functional platforms for next-generation lightweight, energy-absorbing, and smart composite applications.

## Figures and Tables

**Table 1:** Overview of representative reviews and positioning of the present study.

Reference	Focus of the Review/Study	Main Limitations of the Work	Differences in This Review
[1,2,3,5]	Reviews on hybrid polymer composites reinforced with fibers, particulates, or nanofillers.	Focus on polymeric hybridization; no discussion of metallic architectures or load-bearing hybrid systems.	Extends hybridization concepts to continuous metallic architectures (meshes, foams, lattices) and their structural roles.
[4]	Reviews on PMHCs with emphasis on injection overmolding and joining processes.	Process-oriented; limited treatment of topology, architecture, and multifunctional performance.	Integrates architecture–interface–performance relationships, establishing a structural design framework.
[6]	Review of hybrid MMCs.	Focus mainly on metal-dominated systems; lacks polymer–metal synergy analysis.	Positions PMHCs as hybrid composites combining polymer processability with metal strength and conductivity.
[7]	Exploratory analysis of hybrid polymer–metal composite structures (general overview of mechanical and design approaches).	Limited quantitative data and lacks systematic classification by metallic architecture.	Provides a more rigorous typological framework linking architecture, interface, and multifunctional performance.
[11]	Review on surface modification of polymers for improved adhesion.	Material chemistry focus; not specific to hybrid composites or structural implications.	Incorporates surface engineering as part of the multi-scale architecture–interface hierarchy.
[13]	State-of-the-art studies on metallic foams and selected polymer–metal hybrid foam systems.	Single-material system; lacks broad synthesis across architectures.	Expands findings into a cross-architecture framework linking mechanical and functional properties.
[17]	Foundational text on AM technologies.	Broad coverage of AM; lacks application to metal–polymer hybrid architectures.	Connects AM-based topology design to PMHC development, emphasizing hybrid manufacturing.
[18]	Review on electromagnetic riveting and joining techniques for hybrid metal–polymer structures.	Restricted to process-level studies; no consideration of topology, hybrid AM, or multifunctionality.	Integrates joining and surface-engineering methods within an architecture–interface–performance context.
[20]	Review of polymer–metal nanocomposites for electrical and dielectric performance.	Focused on nanoscale systems; lacks connection to macro-architectures and structural hybrids.	Extends the discussion from nanoscale conductive fillers to continuous and architected metallic networks enabling multifunctionality in PMHCs.
[21]	Modeling and cohesive zone approaches for predicting metal–polymer interfacial behavior.	Theoretical; neglects architectural or multi-material geometries.	Connects interfacial modeling to hierarchical design and mechanical functionality in PMHCs.
[22]	Book chapter on polymer–metal hybrid systems for biomedical and functional applications.	Application-driven; lacks comparative synthesis of architectures and interfacial effects.	Shifts the focus from application-driven biomedical systems to an architecture-centered analysis of load-bearing PMHCs.
[23]	Review on 3D-printed polymer–metal scaffolds for biomedical use.	Focus on biocompatibility; limited structural analysis.	Extends insights from biomedical AM scaffolds toward an architecture-driven perspective on load-bearing and multifunctional PMHCs.

**Table 2:** Representative works on PMHCs with continuous metallic reinforcements.

Ref.	Metallic Reinforcement	Study Focus	Main Findings
[7]	Mechanical performance and hybrid structural design	Mechanical performance of hybrid polymer–metal laminates reinforced with wire mesh networks	Showed that embedded wire meshes increase tensile stiffness and load-bearing capacity; provided structural optimization guidelines for hybrid laminates.
[25]	Steel mesh/local metallic inlays	Formability and bending of metal–polymer–metal sandwich composites	Demonstrated that localized mesh reinforcements enhance deep-drawing formability while maintaining global stiffness; optimal mesh positioning minimizes delamination during bending.
[26]	Aluminum mesh (various aperture sizes)	Tensile and interfacial behavior of bamboo fiber/aluminum mesh polymer composites	Revealed that mesh aperture and stacking sequence significantly affect tensile strength and ductility; fine meshes improve interfacial stress transfer and load uniformity.
[27]	Stainless-steel and copper meshes	Electromagnetic shielding and multifunctional PMHCs	Demonstrating simultaneous improvements in EMI shielding effectiveness and flexural stiffness.
[28]	Continuous metallic sheets in hybrid laminates	High strain-rate and impact behavior of polymer–metal hybrids	Found that continuous metallic layers substantially improve energy absorption, impact resistance, and failure tolerance while maintaining lightweight characteristics.

**Table 3:** Representative works on PMHCs with interfacial engineering and bonding mechanisms.

Ref.	Interfacial Engineering and Bonding Mechanisms	Study Focus	Main Findings
[30]	Aluminum–polymer–aluminum sandwich sheets	Development of a new joining method for metal–polymer–metal panels	Introduced a combined mechanical–chemical bonding route doubling interface strength vs. adhesive joints; stable under cyclic loading.
[31]	Mg alloy sheet bonded with CFRP layers	Enhancement of interfacial adhesion in CFRP/Mg hybrid laminates	Surface-treated Mg alloy layers increased interfacial shear strength and reduced delamination; surface chemistry crucial for durable bonding.
[33]	Metal–polymer interface (numerical model)	Finite element simulation of interface failure in metal–composite hybrids	Developed FEM framework predicting crack initiation/propagation; accurate energy release rate predictions validated experimentally.
[34]	Integrated metallic sheets in polymer matrix	Constitutive and cohesive zone modeling of interface behavior	Proposed cohesive model describing adhesive–cohesive transition and failure propagation; improved predictive design for PMHCs.
[35]	Metallic sheet with surface-modified polymer interface	Experimental + numerical study of hybrid interface behavior	In situ monitoring and FEM revealed stress evolution and fatigue damage at interface; clarified mechanisms of crack initiation.
[37]	Mechanical joint-based metal–composite interfaces	Load transfer and interface optimization in mechanical joints	Demonstrated that optimized joint geometry and preload improve load transfer efficiency and delay interfacial failure.
[39]	Metal–polymer sandwich composite sheets	Joining via local mechanical interlocking (“mechanical nuggets”)	Showed that mechanical interlocking yields strong adhesive-free joints with higher fatigue resistance and recyclability.
[32]	Laser joining of polymer–metal hybrid structures	Comprehensive review of laser-based bonding processes	Identified optimal process parameters (power, scan speed) and surface topology effects enabling strong and localized metal–polymer adhesion; highlighted applicability to lightweight manufacturing.

**Table 4:** Representative works on PMHCs with porous and foam-based architectures.

Ref.	Porous and Foam-Based Architectures	Study Focus	Main Findings
[41]	Metal foams: production and stability	Fundamental study on fabrication and microstructural control of metallic foams	Established the relationship between pore morphology, stability, and mechanical behavior; provided key design principles applicable to polymer–metal hybrid foams.
[42]	Aluminum foam–polymer hybrid (APM process)	Development of aluminum foam–polymer composites with controlled pore morphology for lightweight structural applications.	Introduced an advanced pore morphology (APM) approach using adhesive bonding between foam elements; achieved high energy absorption and mechanical stability suitable for automotive structural reinforcement.
[43]	Open-cell aluminum foam infiltrated with thermoplastic polymer	Noise–vibration–harshness (NVH) characterization and damping behavior of interpenetrating metal–polymer composites.	Demonstrated superior damping and vibration attenuation; identified interfacial bonding quality as critical to dynamic performance.
[44]	Aluminum foam–polymer hybrid system	Experimental study on stiffness and damping coefficients for vibration isolation applications.	Reported increased damping and dynamic stiffness; interface modeled as a parallel spring–damper system highlighting interfacial contribution to energy dissipation.
[46]	Polymer–metal interpenetrating phase composite	Finite element modeling (tetrakaidecahedral unit cell) of mechanical response in foam-based PMHCs.	Showed that axial ligaments enhance stiffness, while transverse ligaments induce stress concentration; provided a predictive model correlating foam geometry and mechanical behavior.
[47]	General review of metal foam–polymer hybrids	Overview of fabrication methods (infiltration, adhesive joining) and structure–property relationships.	Summarized processing routes, mechanical trends, and application fields; highlighted foam morphology as the dominant factor controlling energy absorption.
[48]	Cu–melamine foam hybrid	Application of porous metal–polymer scaffolds in bioelectrochemical systems.	Demonstrated durable, conductive, and elastic Cu–polymer foams for microbial fuel cells with long-term electrochemical stability (>75 days).
[49]	Polymer-coated hybrid metallic foams	Fabrication of multifunctional foams with high elasticity, conductivity, and pressure sensitivity.	Achieved hybrid foams combining metal-like conductivity with polymer-like resilience; potential for sensors and flexible electronics.
[50]	MOF–polymer hybrid porous systems (nanoscale, non-structural)	Review of MOF–polymer hybrid materials and interpenetrated architectures.	Provided comprehensive synthesis pathways for porous MOF–polymer systems; emphasized tunable porosity and selective permeability for separation and filtration applications.
[51]	Nanoporous metal–organic frameworks (MOFs)	Biomedical applications of nanoporous polymer–metal composites.	Discussed nanoporous MOF–polymer hybrids with high surface area and chemically tunable porosity, primarily targeting biomedical drug delivery and biosensing rather than load-bearing applications.

**Table 5:** Representative works on PMHCs with lattice- and topology-optimized structures.

Ref.	Lattice- and Topology- Optimized Structures	Study Focus	Main Findings
[19]	Multifunctional polymer–metal lattices via hybrid additive manufacturing	Integration of vat photopolymerization and electroless/electroplating for Ni-P/Cu-coated polymer lattices.	Produced lattice composites with high strength, conductivity, and ductility; demonstrated applicability in UAV structural components.
[53]	Topology, size, and shape optimization of polymer–metal hybrid structural components	Development of computational optimization tools for PMHC automotive body-in-white structures.	Introduced early multi-parameter optimization combining topology, shape, and size for stiffness and manufacturability; established the basis for hybrid lightweight design under service loads.
[55]	Hybrid metal/composite lattice structures for additive manufacturing	Numerical optimization and experimental validation of hybrid lattices fabricated by ALM (metal) and filament winding (composite).	Demonstrated automatic optimization of lattice unit cells via ANSYS–Mode Frontier coupling; improved buckling and load-bearing capacity of hybrid composite–metal structures.
[56]	Topology optimization-guided polymer–metal lattice composites	Design of bioinspired topologically optimized lattices and metallized hybrid composites.	Achieved specific modulus of 5417 MPa·kg^−1^ and energy absorption efficiency of 78%; revealed fracture and deformation mechanisms using FEM and DIC.
[58]	Solid–lattice hybrid architectures for aerospace structures	Multi-scale optimization combining solid and lattice domains for lightweight, high-performance design.	Proposed a two-step topology and lattice optimization framework; achieved 20–30% weight reduction with improved mechanical performance in aerospace components.
[59]	AM compression overmolding of metal–polymer lattices	Experimental fabrication of maraging steel lattices overmolded with carbon fiber-reinforced polyamide.	Demonstrated high stiffness and tensile strength; validated by microscopy and FEM correlation (<20% deviation); introduced new hybrid AM–molding process.
[60]	Topology-optimized polymer lattice beams with perimeter reinforcement	Optimization of composite lattice beams for enhanced post-failure toughness.	Showed that perimeter constraints prevent shear banding and increase post-failure load capacity; validated via 3-point bending tests.
[61]	Topology-optimized novel lattice structures for energy absorption	Application of BESO algorithm for developing CompIED and ShRComp topologies with superior impact resistance.	New lattice topologies exceeded isotropic elasticity limits; exhibited high compression strength and perforation resistance; validated via impact and FEA tests.
[62]	Lattice–stiffener hybrid cores for composite sandwich panels	Multi-material topology optimization for stiffness and uniformity in hybrid core design.	Developed optimization model with penetration constraints; achieved uniform lattice–stiffener distribution and improved load transfer in composite panels.

**Table 6:** Representative works on PMHCs with HAM approaches.

Ref.	Hybrid AM Approaches	Study Focus	Main Findings
[12]	Architected cellular materials and meta-structures	Review of topologically engineered lattices fabricated via AM.	Established design principles for stiffness- and energy-optimized lattices; foundational for HAM-based architected PMHCs.
[18]	3D-printed polymer–metal porous scaffolds	AM of metal–polymer composites with biomedical and structural relevance.	Demonstrated process integration of AM metals and polymers for multifunctional hybrid scaffolds; extended concept to structural PMHCs.
[19]	Hybrid AM combining vat photopolymerization and electroless/electroplating (Ni–P/Cu coatings)	Fabrication of multifunctional polymer–metal lattice composites.	Produced lattices with high strength, electrical conductivity, and ductility; demonstrated multifunctional UAV structures using dual-phase AM processes.
[59]	AM compression overmolding of maraging steel–polymer lattices	Development of a hybrid AM process integrating laser powder bed fusion (metal) with polymer compression overmolding (CF-reinforced PA).	Achieved high stiffness and tensile strength; validated FEM–experiment correlation (<20% deviation); demonstrated strong metal–polymer adhesion and damage tolerance.
[60]	Topology-optimized polymer lattice beams (additive fabrication)	Optimization of polymer composite lattices via anisotropic topology design to control post-failure response.	Introduced perimeter constraints improving post-failure toughness and preventing shear banding; provides insights for hybrid lattice mechanical design.
[61]	Bidirectional Evolutionary Structural Optimization (BESO) for lattice structures	Topology optimization for energy absorption and impact resistance in AM lattices.	Developed CompIED and ShRComp lattices exceeding isotropic elasticity limits; achieved superior compression and impact performance.
[62]	Multi-material topology optimization for lattice–stiffener hybrid cores	Design and optimization of hybrid sandwich panel cores integrating composite and metallic domains.	Achieved uniform lattice–stiffener distribution; enhanced load transfer and stiffness; validated through computational optimization.
[66]	AM-based polymer/metal composite scaffolds (bioprinting and infiltration)	Review on AM of 3D porous polymer–metal scaffolds for biomedical use.	Demonstrated co-fabrication of metal and polymer scaffolds with tunable porosity and mechanical properties; relevant to architected PMHC design.
[63]	General classification of AM and hybrid AM technologies	Foundational reference on AM process categories including hybrid manufacturing.	Provided taxonomy of additive, subtractive, and hybrid systems; basis for classifying hybrid AM in PMHC design.

**Table 7:** Representative works on PMHCs with functional performance.

Ref.	Hybrid System	Functional Focus	Main Findings
[20]	Polymer–metal nanoparticle hybrids	Electrical & dielectric behavior	Analyzed influence of nanoparticle dispersion on conductivity, permittivity, and EMI attenuation in PMHC nanocomposites.
[21]	PANI/graphite-enhanced polymer composites	Electrical & EMI performance	Enhanced electrical conductivity and 72 MPa tensile strength; hybrid graphite–polymer networks enable robust multifunctionality.
[27]	CFRP with metal wire mesh interlayers	EMI shielding & mechanical strength	Embedded Ni/Cu meshes improved EMI SE (20–60 dB) and flexural strength by 25%; strong PMHC analog.
[64]	Polymer–metal–carbon composite review	EMI & heat conduction	Overview of hybrid filler strategies (Ag, Cu, CNT) to balance conductivity, weight, and mechanical performance.
[69]	Natural rubber + acetylene black composites	EMI shielding & electrical conduction	Achieved 20 dB EMI SE with 25% improved tensile properties; sustainable lossy dielectric approach for flexible PMHCs.
[70]	CF/epoxy hybrid with Fe nanoparticles and MnO_2_ interlayers	EMI shielding, thermal, and mechanical synergy	Interface–layup synergy achieved 70 dB EMI SE, 4.4 W/mK thermal conductivity, and 70 MPa ILSS; exemplary multifunctional PMHC.
[71]	3D metal–polymer network composites	EMI shielding & thermal conduction	3D interconnected metallic/carbon skeletons enhance both EMI SE (>40 dB) and thermal conductivity (>6 W/mK); tunable multifunctionality.
[72]	Fe–Si–Al alloy–polymer hybrid composites	EMI shielding & thermal management	Surface-modified Fe–Si–Al fillers achieved dual functionality: high EMI SE (48 dB) and thermal conductivity (6 W/mK).
[73]	Cu hollow bead–epoxy lightweight composites	EMI shielding & heat dissipation	Achieved ultralight composites (ρ ≈ 1 g/cm^3^) with EMI SE > 100 dB and thermal conductivity up to 7 W/mK; benchmark performance.
[74]	SiO_2_ microparticles + graphene nanofillers in polymer matrix	Thermal conductivity modeling	Micromechanical modeling predicted 3× thermal conductivity increased through hybrid micro/nanofiller coupling.
[75]	Computational study on filler volume effects	Thermal conduction	Demonstrated excluded-volume effects improving filler percolation and increasing thermal conductivity by 20%.
[76]	Review on high-thermal-conductivity polymers	Thermal performance	Identified interfacial alignment and hybrid network engineering as key to achieving >10 W/mK in polymer–metal systems.
[77]	Polymer–metal/carbon hybrid composites	EMI shielding (broadband)	Comprehensive review highlighting synergy between conductive, magnetic, and dielectric mechanisms in PMHCs for EMI mitigation.
[79]	CNT/Fe_3_O_4_/PP phase-change hybrid composites	Thermal management & EMI	Flexible, heat-storing PMHCs with 41 dB EMI SE; improved thermal control under variable conditions.
[80]	PPS composites with CNT/CF/GNP fillers	EMI shielding & thermal conduction	Dual enhancement: EMI SE 50–68 dB and k = 7 W/mK; highlights hybrid filler synergy in PPS-based PMHCs.
[81]	Review of hybrid polymer composites	Electrical, thermal & mechanical	Comprehensive review on the filler architecture and AM-based hybridization for multifunctional performance.
[82]	TPS + waste iron filings composite	Electrical conductivity (sustainable PMHC)	Demonstrated formation of conductive pathways in biodegradable polymer matrix using metallic waste fillers; highlights circular-economy potential.

**Table 8:** Extended classification of metallic architectures in PMHCs.

Metallic Architecture	Load Transfer & Stiffness	Energy Absorption	Damping/ NVH	Electrical & Thermal	EMI Shielding	Manufacturability
Metal meshes & perforated sheets	●●●	●●○	●○○	●●○	●●●	●●●
Continuous metal sheets/laminates	●●●	●●○	●○○	●●●	●●●	●●●
Metal foams & porous scaffolds	●●○	●●●	●●●	●●○	●●○	●●○
Wire networks & woven metal inserts	●●○	●●○	●●○	●●○	●●○	●●○
AM lattices & topology-optimized structures	●●●	●●●	●●○	●●○	●●○	●○○
Metallized/coated polymer architectures	●○○	●○○	●○○	●●●	●●●	●●○

Scale → ●○○ = low|●●○ = medium|●●● = high.

**Table 9:** Representative works on metal architectures in PMHCs.

Category	Structural Characteristics	Functional/Role	Representative References
1. Metal meshes and expanded sheets	2D networks of perforated or woven metallic layers (meshes, expanded foils) embedded in polymers.	In-plane stiffness, crack bridging, improved formability, enhanced delamination resistance.	Woven metallic mesh polymer composites with enhanced tensile strength and ductility [59]; hybrid MPM sandwich composites showing improved forming and bending behavior [24]; expanded metal mesh-based hybrid laminates with improved mechanical and environmental performance [85].
2. Metal foams and porous scaffolds	Open- or closed-cell 3D metallic foams (random porosity), typically polymer-infiltrated.	Energy absorption, damping, lightweight design.	AM lattice hybrids with optimized topology and buckling resistance [12,55]; fundamental works on foam morphology, porosity, and mechanical stability [13,41].
3. Wire networks and woven metallic fabrics	Interlaced wires, knitted/braided metallic structures (woven/knitted fabrics).	Flexibility, anisotropy, high fatigue tolerance.	Patterned inserts and laser-textured Mg/epoxy hybrids showing strong mechanical interlocking [30,31]; woven metallic mesh polymer composites with enhanced tensile strength and ductility [59].
4. Lattice and cellular structures	Periodic or graded 3D lattices fabricated by AM.	Controlled topology, stiffness-to-weight optimization, multifunctionality (structural–functional integration).	BESO-designed hybrid lattices and composite sandwich cores [61,62]; AM lattice hybrids with optimized topology and buckling resistance [12,55]; multi-material topology optimization approaches for lightweight hybrid structures [86].
5. Perforated or patterned metallic inserts	Sheets or plates with laser-drilled holes, embossed textures, or ribs (patterned/perforated inserts).	Mechanical interlocking, enhanced polymer infiltration, improved interfacial adhesion.	Graded metal–polymer lattices with tunable mechanical and thermal response [58,59]; patterned inserts and laser-textured Mg/epoxy hybrids showing strong mechanical interlocking [30,31].
6. Hierarchical or hybrid architectures	Hierarchical or multi-scale combinations (e.g., lattice core + mesh skin; multi-layer hybrids).	Multi-scale stress distribution, high energy absorption, improved impact resistance (progressive failure).	Ni–P/Cu-coated polymer lattices with multifunctional performance [19,23]; BESO-designed hybrid lattices and composite sandwich cores [61,62]; comparative analyses of hybrid architectures (FML vs. MMC) highlighting multi-scale design principles [87].
7. Gradient and functionally graded metallic architectures (FGM)	Functionally graded metallic architectures with continuously varying density/porosity (or composition) through thickness.	Stress tuning, local stiffness control, tailored stress distribution, thermal management.	Experimental and modeling studies on interpenetrating hybrid foams [43,46]; graded metal–polymer lattices with tunable mechanical and thermal response [58,59].
8. 3D-printed metallic coatings/metallized skeletons	Thin metallic coatings on polymer scaffolds (electroplating, vapor deposition; metallized lattices).	Electrical/thermal conductivity, EMI shielding, corrosion protection.	Surface texturing and laser joining enhancing polymer–metal interface quality [11,32]; Ni–P/Cu-coated polymer lattices with multifunctional performance [19,23].
9. Interpenetrating metal–polymer networks (IPNs)	Interpenetrating metal–polymer networks (both phases continuous; fully infiltrated porous metals).	Synergistic ductility + stiffness, vibration damping, improved damage tolerance.	Woven metallic mesh polymer composites with enhanced tensile strength and ductility [59]; experimental and modeling studies on interpenetrating hybrid foams [43,46]; damage evolution in fiber metal laminates highlighting architecture-dependent crack propagation [88]; experimental studies on fracture propagation and interface-driven damage mechanisms in hybrid laminates [89].
10. Micro-textured or surface-engineered metallic layers	Micro-/nanostructured or surface-engineered metals (laser, plasma, anodic oxidation, chemical treatments).	Adhesion improvement, interface durability (fatigue/environmental), hybrid bonding robustness.	AM lattice hybrids with optimized topology and buckling resistance [12,55]; surface texturing and laser joining enhancing polymer–metal interface quality [11,32].

## Data Availability

No new data were created or analyzed in this study. Data sharing is not applicable to this article.

## Abbreviations

The following abbreviations are used in this manuscript:

AFSP	Additive Friction Stir Processing
AM	Additive Manufacturing
APM	Advanced Pore Morphology
BESO	Bidirectional Evolutionary Structural Optimization
CF	Carbon Fiber
CFRP	Carbon Fiber Reinforced Polymer
CNT	Carbon Nanotubes
CZM	Cohesive Zone Model
DED	Directed Energy Deposition
DIC	Digital Image Correlation
EBM	Electron Beam Melting
EMI	Electromagnetic Interference
FE/FEM	Finite Element/Finite Element Method
FFF/FDM	Fused Filament Fabrication/Fused Deposition Modeling
FRPs	Fiber-Reinforced Polymers
GNPs	Graphene Nanoplatelets
HAM	Hybrid Additive Manufacturing
ILSS	Interlaminar Shear Strength
LPBF	Laser Powder Bed Fusion
MMCs	Metal Matrix Composites
MnO_2_	Manganese Dioxide
MOF	Metal–Organic Framework
Ni–P	Nickel–phosphorus Coating
PA/PANI	Polyamide/Polyaniline
PEEK	Poly(ether ether ketone)
PMHCs	Polymer–Metal Hybrid Composites
PP/PPS	Polypropylene/Polyphenylene sulfide
PRISMA	Preferred Reporting Items for Systematic Reviews and Meta-Analyses
SE	Shielding Effectiveness
SiO_2_	Silicon Dioxide
σ	Electrical Conductivity
ρ	Density
k	Thermal Conductivity
